# Route of Injection Affects the Impact of InlB Internalin Domain Variants on Severity of* Listeria monocytogenes* Infection in Mice

**DOI:** 10.1155/2017/2101575

**Published:** 2017-12-28

**Authors:** Konstantin A. Sobyanin, Elena V. Sysolyatina, Yaroslava M. Chalenko, Egor V. Kalinin, Svetlana A. Ermolaeva

**Affiliations:** Gamaleya Research Center of Epidemiology and Microbiology, Moscow 123098, Russia

## Abstract

The facultative intracellular pathogen* Listeria monocytogenes* causes a severe food-borne infection in humans and animals.* L. monocytogenes* invasion factor InlB interacts with the tyrosine kinase c-Met via the N-terminal internalin domain. Previously, distinct variants of the InlB internalin domain (idInlB) have been described in* L. monocytogenes* field isolates. Three variants were used to restore full-length InlB expression in the* L. monocytogenes* strain EGDeΔ*inlB*. Obtained isogenic* L. monocytogenes* strains were tested in the invasion assay and intravenous, intraperitoneal, and intragastric models of infection in mice. All idInlBs were functional, restored InlB activity as an invasion factor, and improved invasion of the parental strain EGDeΔinlB into human kidney HEK23 cells. Meanwhile, distinct idInlBs provided different mortality rates and bacterial loads in internal organs. When recombinant strains were compared, the variant designated idInlB14 decreased severity of disease caused by intravenous and intraperitoneal bacterial administration, whereas this variant improved intestine colonization and stimulated intragastric infection. Obtained results demonstrated that naturally occurring idInlBs differed in their impact on severity of* L. monocytogenes* infection in mice in dependence on the infection route.

## 1. Introduction

The Gram-positive bacterium* Listeria monocytogenes* is a causative agent of food-borne infection, listeriosis, in humans and many domestic and wild animals [1, 2]. Listeriosis is a rare but severe infection with lethality reaching 30% in humans [3, 4]. The most serious clinical manifestations of listeriosis are CNS disorders and fetus impairment in pregnant females.

The intestine is a port of bacterial entry in the course of food-borne listeriosis [1, 5].* L. monocytogenes *crosses the intestinal barrier and colonizes the lamina propria from where it spreads to internal organs [5]. Systemic listeriosis includes colonization of the liver and spleen. If the infection is not controlled at this stage, a secondary bacteremia develops, and bacteria cross cerebral and/or maternal-fetal barriers to infect the brain and fetus [1, 2, 5, 6].

*L. monocytogenes* is a facultative intracellular pathogen that infects macrophages and a wide range of nonprofessional phagocytes [7]. Internalin (InlA) and InlB are major factors that provide active invasion in nonprofessional phagocytes [7, 8]. InlA and InlB belong to the internalin family. Internalin family proteins include an internalin domain, which consists of a central leucine rich repeat- (LRR-) domain flanked by amino-terminal N-cap and carboxy-terminal immunoglobulin-like (Ig-like) domains [9]. The internalin domain is directly involved in specific protein-protein interactions with mammalian cell surface receptors. InlB internalin domain specifically interacts with the eukaryotic receptor c-Met/HGFR (Hepatocyte Growth Factor Receptor) [10].

Mouse models are of key importance for studies of* L. monocytogenes* virulence. LD50 and bacterial counts in the liver and spleen are major ways used for assessment of* L. monocytogenes* strain virulence [11, 12]. Intravenous (i.v.) and intraperitoneal (i.p.) infection routes usually require a smaller infective dose and are used more often in* L. monocytogenes* research than intragastric (i.g.) or oral routes [13–15]. When several strains were compared, their relative virulence was dependent on the route of infection. Certain strains highly virulent when injected i.p. or i.p. are less virulent when applied orally or i.g., and vice versa [16, 17].

*L. monocytogenes* oral route infection in mice is dependent on InlB, which accelerates listerial invasion into M cells of ileal Peyer's patches (PPs) [18, 19]. Previously, we and others described isolation of* L. monocytogenes* from internal organs of wild small rodents belonging to the* Murinae* subfamily, which is closely related to* Mus musculus*. These data suggest that mouse intestine barrier crossing is effective enough for systemic infection to be established in the natural environment [20, 21]. Among 15 InlB variants found in* L. monocytogenes* strains, only two variants were revealed in* L. monocytogenes* isolated from wild small rodents [21, 22]. Existence of specific InlB variants was demonstrated for* L. monocytogenes* strains characterized by reduced virulence [23] or associated with cardiovascular disease [24].

To evaluate an impact of naturally occurring InlB variants on* L. monocytogenes* virulence, we used a set of previously described isogenic strains that carried distinct InlB internalin domains (idInlBs) [25]. Virulence of the recombinant strains was compared in mouse models. Distinct idInlBs provided different mortality rates and bacterial loads in internal organs, and the difference was dependent on the route of infection.

## 2. Materials and Methods

### 2.1. Bacterial Strains and Growth Conditions

Bacterial strains used in the work are listed in Table 1.* L. monocytogenes* were routinely cultivated in the Brain Heart Infusion (BHI, BD, USA) medium. To get a virulence regulon induction,* L. monocytogenes* was grown in BHI supplemented with 0,2% activated charcoal (Merck) [26].* E. coli* used in cloning and expression procedures were cultivated in the LB medium (Sigma-Aldrich). All bacteria were grown at 37°C with agitation at 180 rpm. Antibiotics were added up to the following concentrations: erythromycin up to 10 and 300 *μ*g ml^−1^ for* L. monocytogenes* and* E. coli*, respectively; ampicillin up to 100 *μ*g ml^−1^; kanamycin up to 200 *μ*g ml^−1^. All antibiotics were purchased from Sigma-Aldrich. To prepare a culture for infection, bacteria were grown up to mid-exponential phase, washed with PBS, aliquoted, and frozen in the presence of 10% glycerin. The concentration was determined by plating serial dilutions from the frozen culture the day before the experiment. Bacteria were thawed immediately before the experiment and resuspended in PBS up to the required concentration.

### 2.2. Mammalian Cells and Cultivation Conditions

Human embryonic kidney HEK-293 cells (the public collection of Gamaleya Center; http://virology.gamaleya.org/index.php?option=com_content&view=article&id=103:kollektsiya-kletochnykh-kultur&catid=91&Itemid=645) were used. Cells were grown in the DMEM medium supplemented with 10% FBS (fetal bovine serum) in the 5% CO_2_ atmosphere. In some experiments FBS was decreased up to 2%.

### 2.3. Recombinant Strains

Recombinant strain construction was described previously [25]. Shortly, the idInlB encoding DNA fragments obtained on lysates of* L. monocytogenes* strains VIMHA004, VIMHA015, and VIMHA034 (Table 1) with primers InlB3: 5′-CAAGCG**GGATCC**ATCACCGTGCCAACGCC and InlB4: 5′-TATCCG**GGATCC**TGCTTCTACTTTTG were cloned into the derivative of the pTRKH2 vector [29] carrying the* inlB* gene, lacking the internalin domain encoding part. The obtained plasmids encoded the full-length InlB with different idInlBs. The plasmids were incorporated into the* L. monocytogenes* EGDeΔinlB strain by electroporation.

### 2.4. Cell Invasion Assay

Bacteria were prepared from the mid-exponential culture as it was described above. The “gentamycin assay” was performed as described previously [25]. Briefly, bacteria resuspended in DMEM were added with MOI 100 : 1 (bacteria to cell) to cells, incubated 1 h at 37°C in 5% CO_2_ atmosphere, and washed with PBS, and then the fresh medium supplemented with 100 *μ*g ml^−1^ gentamycin (Fluka) was added. In 1 h, cells were washed and lysed with 1% Triton X-100. Serial dilutions of cell lysates were plated to count bacterial colonies. The invasion efficiency was determined as the ratio of intracellular bacteria to the number of applied bacteria.

### 2.5. Mice

Experiments on animals were conducted in accordance with the Russian Federation National Standard (GOST R52379-2005), directives of Ministry of Health of Russian Federation (number 753n from 26.08.2010; number 774н from 31.08.2010), and with the approval of the Biomedical Ethics Committee of Gamaleya Research Center of Epidemiology and Microbiology (number 93 from 22.10.2015). Animals were sacrificed using ether inhalation. Female BALB/c mice of 16–18 g, purchased from the nursery Stolbovaya (Moscow region, Russia), were kept at our breeding facilities in specific-pathogen-free conditions and used in all experiments.

### 2.6. Experimental Infection in Mice

For intravenous infection, mice were prewarmed with air heater and injected into the tail vein with bacteria suspended in 100 *μ*l PBS. The doses are indicated in the text. For intraperitoneal infection, mice were injected intraperitoneally with bacteria suspended in 200 *μ*l PBS. For intragastric infection, mice starved for 12 h were anesthetized by intraperitoneal injection of sodium pentobarbital (40 *μ*g/g) and infected with bacteria suspended in 200 *μ*l PBS by using the needle with the diameter 0.6 mm and the length 25 mm. The tip of the needle was cut and polished to smoothness to avoid stomach injury.

### 2.7. Mortality Rates and Bacterial Loads in Organs

To determine mortality rates, groups of 20 animals were infected i.v. with doses of bacteria higher or near LD50. The experiment was stopped as soon as 50% lethality was observed in at least one of the groups and all remained animals were euthanized.

Groups of 5 animals were used to determine bacterial loads in infected organs. Animals were euthanized with ether at time points specified in the text. The liver and the spleen were aseptically harvested and homogenized in 1 and 0,2 ml sterile PBS, respectively. The intestine was washed out with sterile PBS; visible Peyer's patches were separated and homogenized in 5 ml of sterile PBS. Small intestine was separately homogenized in 5 ml of sterile PBS. Tenfold serial dilutions of organ homogenates were plated on BHI agar supplemented with antibiotics if required. Plates were incubated at 37°C for 24 h The detection limit of this procedure was 10, 10, and 10^2^ colony forming units (CFU) per liver, spleen, and intestine, respectively.

### 2.8. Statistics

All experiments were performed using duplicate samples. Results of repeated experiments were averaged. The one-tailed *t*-test was used for assessment of statistical significance.

## 3. Results

### 3.1. idInlB Variants Differentially Supported i.v.* L. monocytogenes* Infection

**Figure 1:**
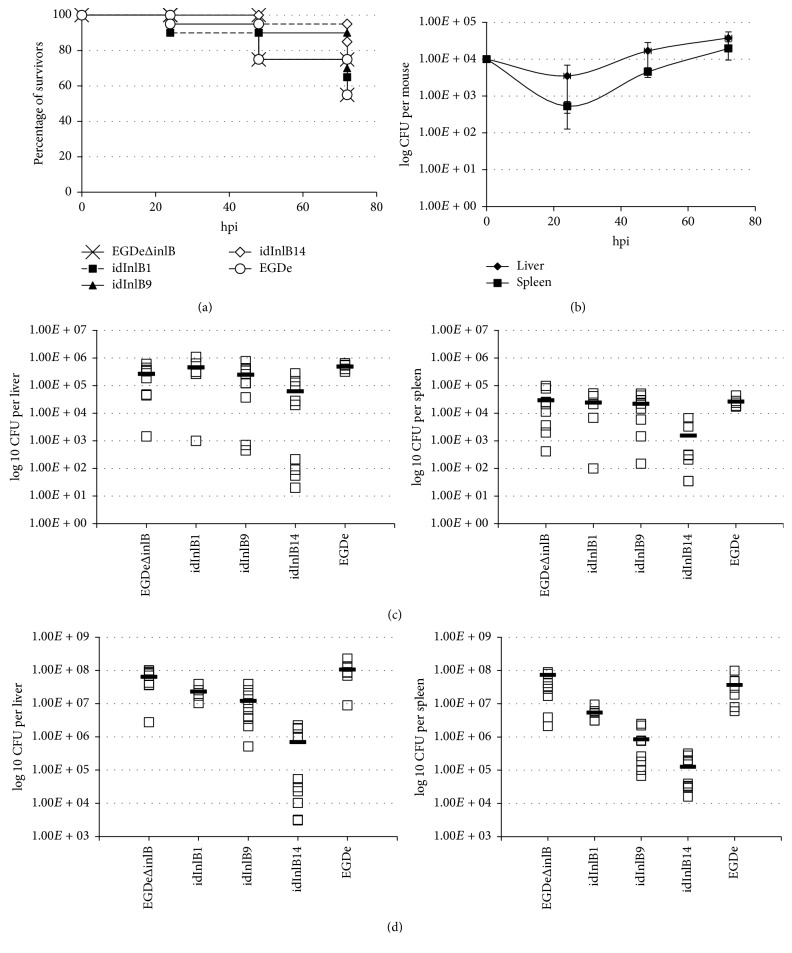
Impact of idInlB variants on severity of intravenous infection in BALB/c mice. (a) Mortality caused by parental EGDeΔinlB, wild-type EGDe, and recombinant EGDeΔinlB::InlB1 (idInlB1), EGDeΔinlB::InlB9 (idInlB9), and EGDeΔinlB::InlB14 (idInlB14) strains. Groups of 20 mice were infected i.v. with 2 × 10^6^ CFU as described in Materials and Methods. (b) Proliferation of EGDeΔinlB::InlB14 in the liver and spleen of mice i.v. infected with 1 × 10^4^ CFU. Average and SD for groups of 5 mice are shown. (c) and (d) idInlB-dependent drop of bacterial loads in the liver and spleen 6 (c) and 24 (d) hpi. BALB/c mice were i.v. infected with 1 × 10^6^ CFU. The data and average of two independent experiments performed on groups of 5 mice are shown.

Firstly, we evaluated an impact of idInlB variants on the final outcome of intravenous (i.v.) infection. To compare mortality rates, 2 × 10^6^ CFU of parental EGDeΔinlB, wild-type EGDe, or each recombinant strain was injected into the tail vein of female BALB/c mice. This bacterial load was near LD50 for the parental strain EGDeΔinlB (LD50 ≈ 1 × 10^6^ in our experimental settings) and much higher than LD50 for the wild-type strain EGDe (LD50 ≈ 1,5 × 10^4^). The 50% mortality was reached in 72 h after infection with both EGDeΔinlB and the wild-type strain EGDe (Figure 1) that was in line with the high infection dose. Mortality caused by all but one recombinant strains had similar rates. However, bacteria carrying idInlB14 caused noticeably lower mortality rates with only 5% mortality during the first three days (Figure 1(a)).

To understand, whether the strain EGDeΔinlB::InlB14 was able to multiply within the host we followed bacterial loads in the liver and spleen of mice infected with 10^4^ bacteria of this strain. A noticeable drop in bacterial loads was observed 24 hpi in comparison with the dose applied for infection: only (3,5 ± 2,3) × 10^3^ CFU per mouse were found in the liver and (5,3 ± 1,8) × 10^2^ CFU per mouse in the spleen (Figure 1(b)). However, after the initial drop, bacterial loads increased logarithmically suggesting that the strain is competent to multiply within the host.

To check whether the same drop happens with all other strains and at high infection doses and to establish at what time point it takes place, we compared bacterial loads in the liver and spleen for all strains 6 and 24 h after i.v. infection with 10^6^ bacteria. By 6 hpi, the number of wild-type EGDe bacteria was higher than EGDeΔinlB in both the liver (4,92 × 10^5^ versus 2,7 × 10^5^ CFU per mouse, resp., *p* < 0,05) and the spleen (2,97 × 10^4^ versus 2,4 × 10^4^ CFU per mouse). The recombinant strain EGDeΔinlB::inlB9 behaved similarly to the parental, with average values of 2,47 × 10^5^ and 2,20 × 10^4^ CFU per liver and spleen, respectively. The strain EGDeΔinlB::inlB1 behaved more closely to the wild-type strain (4,57 × 10^5^ and 2,4 × 10^4^ CFU per liver and spleen, resp.). The strain EGDeΔinlB::inlB14 demonstrated the lowest loads in both the liver and spleen (6,25 × 10^4^ and 1,25 × 10^3^ CFU per liver and spleen, resp., *p* < 0,05).

By 24 hpi, EGDe and EGDeΔinlB accumulated in the liver to 1,08 × 10^8^ and 6,47 × 10^7^ CFU per mouse, respectively, while EGDeΔinlB::InlB1 and EGDeΔinlB::InlB9 accumulated to slightly lower values (2,32 × 10^7^ and 1,22 × 10^7^ CFU per mouse, resp., *p* < 0,05; Figure 1(d)). The difference between EGDeΔinlB and EGDeΔinlB::InlB14 reached 2 log⁡10 (*p* < 0,01). In the spleen, all recombinant strains accumulated to values noticeably lower than control bacteria (*p* < 0,05). The difference between EGDeΔinlB::InlB14 and the parental strain in the spleen reached 2,76 log⁡10 (7,42 × 10^7^ and 1,27 × 10^5^ CFU per mouse for EGDeΔinlB and EGDeΔinlB::InlB14, resp., *p* < 0,01).

Taken together, obtained results demonstrated that (i) InlB hyperproduction rather worsened than improved bacterial accumulation in the first 24 h; this effect was especially noticeable in the spleen 24 hpi; and (ii) InlB internalin domain variants differed in their ability to support i.v. infection with the idInlB14 demonstrating the worst results.

### 3.2. idInlBs Differed in Their Ability to Support i.p. Infection

**Figure 2:**
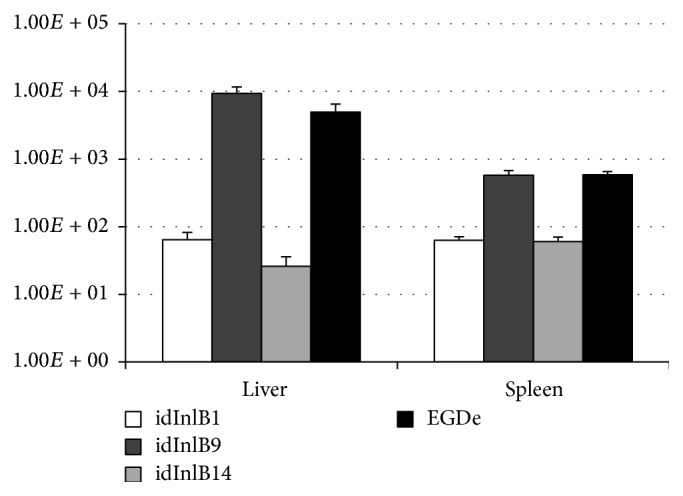
Impact of id InlB variants on severity of intraperitoneal infection in BALB/c mice. Results are shown as a relative increase of bacterial loads in the liver and spleen for the recombinant and wild-type strains comparatively with the strain EGDeΔinlB which lacked the inlB gene. The strains are designated as follows. EGDeΔinlB::InlB1, idInlB1; EGDeΔinlB::InlB9, idInlB9; EGDeΔinlB::InlB14, idInlB14; and EGDe, wild-type strain EGDe.

In contrast with i.v. infection, all recombinant strains had improved bacterial loads in comparison with the parental strain EGDeΔinlB, when 10^6^ bacteria were injected intraperitoneally (i.p.) and bacterial loads in the liver and spleen were detected 72 hpi (Figure 2; *p* < 0,05). However, pairwise strain comparison demonstrated that the EGDeΔinlB::InlB9 supplied noticeably higher infection levels than the variants EGDeΔinlB::InlB1 and EGDeΔinlB::InlB14. The effect was more pronounced in the liver: recombinant strain counts were higher comparatively to the parental (9306 ± 2342)-fold for EGDeΔinlB::InlB9 and (64 ± 18)- and (26 ± 6)-fold for EGDeΔinlB::InlB1 and EGDeΔinlB::InlB14, respectively. Loads of the wild-type EGDe strain were (4954 ± 1582)-higher than EGDeΔinlB. The same tendency was observed for colonization of the spleen: the counts of EGDeΔinlB::InlB9 and wild-type bacteria exceeded the counts of the parental strain (577 ± 97)- and (586 ± 70)-fold, respectively, while the counts of EGDeΔinlB::InlB1 and EGDeΔinlB::InlB14 exceeded parental (63 ± 8)- and (60 ± 11)-fold only.

### 3.3. idInlB9 and idInlB14 Differentially Support Intestine Colonization

Previously, we have demonstrated that the idInlBs differed in their ability to support intragastric (i.g.) infection providing different bacterial loads in the liver but not in the spleen [25]. The idInlB14 gave rise to the highest loads in the liver 3 days after i.g. inoculation. In contrast, idInlB9, which as we demonstrated here provided high bacterial loads after i.p. injection, was low efficient in i.g. route infection [25].

**Figure 3:**
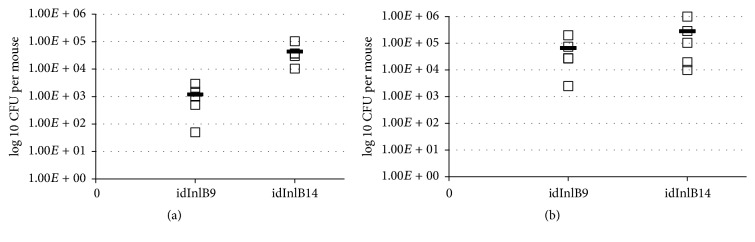
Impact of id InlB variants 9 and 14 on colonization of the intestine. Bacterial loads in Peyer's patches (a) and villous epithelium (b) were determined for groups of 5 BALB/c mice infected intragastrically with 10^8^ CFU of EGDeΔinlB::InlB9 (idInlB9) and EGDeΔinlB::InlB14 (idInlB14).

The internal organ penetration after i.g. infection is not an instantaneous event as it happens after i.v. injection. Colonization of the intestine was shown to play an important role upon i.g. infection [30]. To check whether distinct idInlBs differentially affect gut colonization, EGDeΔinlB::InlB9 and EGDeΔinlB::InlB14 strains were applied i.g. in the dose of 10^8^ CFU per mouse and the number of bacteria in the villous epithelium and Peyer's patches was counted 24 hpi. To count both intracellular and extracellular bacteria associated with the intestine, the intestine was washed thoroughly but it was not treated with gentamycin so both intracellular and surface attached bacteria could be counted. Counting of EGDeΔinlB::InlB14 bacteria was about 1,5log⁡10 higher than EGDeΔinlB::InlB9 bacteria in Peyer's patches (Figure 3; *p* < 0,05). The difference between strains for villous epithelium was not so high, about 3-fold (not significant).

Taken together, obtained results demonstrated that InlB internalin domain variants differed in their ability to support infection in dependence on the route of bacterial injection under otherwise equal conditions. The idInlB14 was less effective when bacteria were injected i.v. while idInlB9 was less effective upon i.g. infection. The third variant idInlB1 demonstrated medium results in all tests.

### 3.4. All idInlBs Provided Invasion in Epithelial Cells

**Figure 4:**
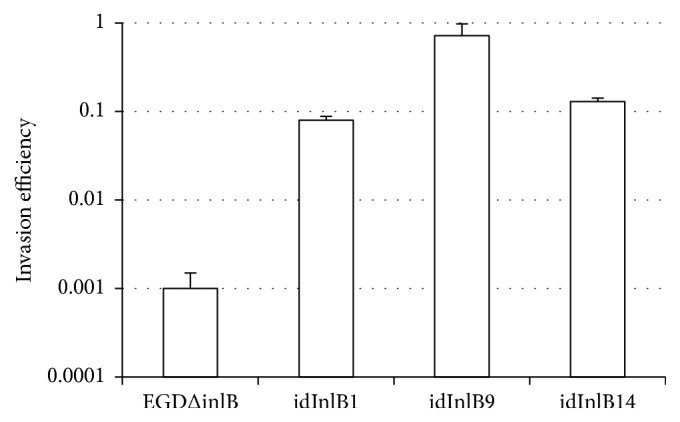
Impact of id InlB variants on invasion efficiency in human HEK293 epithelial cells. Cells were infected with MOI 100 and the invasion efficiency is shown as a ratio of intracellular bacteria to the number of bacteria used for infection. The average and SE of at least 3 independent experiments performed in duplicate are shown.

InlB promotes* L. monocytogenes* invasion in epithelial cells. To check whether idInlBs differ in their ability to support bacterial invasion in epithelial cells, human kidney carcinoma HEK293 cells were infected with recombinant and control bacteria (MOI 100 : 1, bacteria : cells). Relative invasion of EGDeΔinlB::InlB9 was 2,7-fold higher in HEK293 than EGDeΔinlB::InlB14 (Figure 4). However, the difference between recombinant strains was minor in comparison with invasion of the parental strain EGDeΔinlB that was about 2log⁡10 lower for both cell lines. Therefore, all idInlBs were functional to support invasion in epithelial cells.

## 4. Discussion

Mouse models are of key importance for studies of listeriosis.* L. monocytogenes *strains differ considerably in their virulence [11, 12]. Moreover, an infection route affects severity of* L. monocytogenes* infection. The intragastric (i.g.) route is usually noticeably less effective than intravenous (i.v.) and intraperitoneal (i.p.) routes with LD50 differed by 4–6log⁡10 [30, 31]. Still, other works reported a smaller difference within 1-2log⁡10 or a better effectiveness of the i.g. infection when alternative* L. monocytogenes* strains were used [16, 17]. Isolation of* L. monocytogenes* from the internal organs of wild small rodents captured in the pristine habitats further supports the view that systemic* L. monocytogenes* infection in mice might take place via the oral route even at low doses of infection that might be expected in natural ecosystems [20, 22, 32].

Previously, we suggested that the sequence of the invasion factor InlB might be critical for* L. monocytogenes* spreading among small rodents in the pristine environment [21, 25]. Indeed, InlB is a key invasion factor responsible for the intestine barrier crossing in mice [19]. Among 15 InlB internalin domain variants described in* L. monocytogenes* isolated from different hosts, only two variants were discovered in strains isolated from wild small rodents [21, 25]. These are the InlB internalin domain variant 1 designated here the idInlB1, which was found in strains belonging to the phylogenetic lineage I, and the variant idInlB14 found in the lineage II strains. Comparison with available data established that idInlB14 was found in the widely used type strains 10403 and EGD but not in EGDe which was used in this work as a control and which carries the distinct idInlB [21, 25, 33]. Besides listed strains, idInlB14 was described by Témoin et al. in a group of strains that demonstrated low virulence in i.v. infected mice [23]. Complementation of low virulent strains with the plasmid encoding InlB from the strain EGDe improved virulence on the i.v. infection model [23]. Our data are in line with these results demonstrating that idInlB14 caused a decrease in bacterial counts after i.v. infection (see Figure 2). Meanwhile, the idInlB14 variant effectively supported the intragastric infection [25] and intestine colonization (see Figure 3).

## 5. Conclusions

Obtained results demonstrated that naturally occurring InlB internalin domain variants differed in their impact on the outcome of* L. monocytogenes* infection. The impact of distinct idInlBs was determined by the route of infection. The results supported the view that* L. monocytogenes* virulence on the models of intravenous and intraperitoneal mouse infection is not fully consistent with virulence on the intragastric route infection model. Particular idInlB variants might be responsible for this inconsistency.

## Figures and Tables

**Table 1:** Strains and plasmids used in the work.

Species/strain	Characteristics	Reference
*L. monocytogenes*		
EGDe^1^	Serovar 1/2a, type strain	[27]
EGDeΔinlB^1^	EGDe derivative with inlB gene deletion	[28]
VIMHA004	Serovar 4b, ST2^2^, clinical isolate obtained from the stillborn, carried idInlB1^3^	[21]
VIMHA015	Serovar 4b, ST1^2^, Clinical isolate obtained from the stillborn, carried idInlB9^3^	[21]
VIMHA034	Serovar 1/2a, ST314^2^, Clinical isolate obtained from the stillborn, carried idInlB14^3^	[21]
EGDeΔinlB::InlB1	EGDeΔinlB supplemented with the pInlB1 plasmid	[25]
EGDeΔinlB::InlB9	EGDeΔinlB supplemented with the pInlB9 plasmid	[25]
EGDeΔinlB::InlB14	EGDeΔinlB supplemented with the pInlB14 plasmid	[25]
*E. coli*		
JM109	endA1 glnV44 thi-1 relA1 gyrA96 recA1 mcrB+Δ(lac-proAB) e14-[F′ traD36 proAB+lacIqlacZΔM15] hsdR17(rK-mK+)	Promega
BL21 (DE3)	F–ompT gal dcm lon hsdSB(rB-mB-) *λ*(DE3 [lacI lacUV5-T7 gene 1 ind1 sam7 nin5])	NewEngland BioLabs
Plasmids		
pTgem-easy	Cloning vector	Promega
pTRKH2	Shuttle-vector	[29]
pInlAB	pTRKH2::Promoter_inlAB_::gwdInlB^4^	[25]
pInlB1	pInlAB::idInlB1	[25]
pInlB9	pInlAB::idInlB9	[25]
pInlB14	pInlAB::idInlB14	[25]

^1^The strain was generously provided by Professor J. A. Vazquez-Boland, University of Edinburgh. ^2^Sequence tyes (STs) are provided according to the MLST protocol by Ragon et al., 2008. ^3^idInlB is an internalin domain of InlB. ^4^gwdInlB is InlB GW-domain.
